# Multifaceted Pharmacological Potentials of Curcumin, Genistein, and Tanshinone IIA through Proteomic Approaches: An In-Depth Review

**DOI:** 10.3390/cancers15010249

**Published:** 2022-12-30

**Authors:** Farheen Badrealam Khan, Parul Singh, Yahya F. Jamous, Syed Azmal Ali, Abdullah, Shahab Uddin, Qamar Zia, Manoj Kumar Jena, Mohsina Khan, Mohammad Owais, Chih Yang Huang, Venkatesh Chanukuppa, Chrismawan Ardianto, Long Chiau Ming, Waqas Alam, Haroon Khan, Mohammad Akli Ayoub

**Affiliations:** 1 Department of Biology, College of Science, The United Arab Emirates University, Al Ain 15551, United Arab Emirates; 2 Cell Biology and Proteomics Lab, Animal Biotechnology Center, ICAR-NDRI, Karnal 132001, India; 3 King AbdulAziz City of Science and Technology (KACST), Riyadh 12354, Saudi Arabia; 4 Department of Pharmacy, University of Malakand, Chakdara 18800, Pakistan; 5 Translational Research Institute and Dermatology Institute, Academic Health System, Hamad Medical Corporation, Doha 3050, Qatar; 6 Laboratory of Animal Center, Qatar University, Doha 2731, Qatar; 7 Health and Basic Science Research Centre, Majmaah University, Majmaah 11952, Saudi Arabia; 8 Department of Medical Laboratory Sciences, College of Applied Medical Sciences, Majmaah University, Majmaah 11952, Saudi Arabia; 9 Department of Biotechnology, School of Bioengineering and Biosciences, Lovely Professional University, Phagwara 144411, India; 10 Department of Psychiatry, Icahn School of Medicine, Mount Sinai, NY 10029, USA; 11 Interdisciplinary Biotechnology Unit, Aligarh Muslim University, Aligarh 202002, India; 12 Department of Biotechnology, Asia University, Taichung 404, Taiwan; 13 Graduate Institute of Biomedical Sciences, China Medical University, Taichung 404, Taiwan; 14 Cardiovascular and Mitochondrial Related Disease Research Center, Hualien Tzu Chi Hospital, Buddhist Tzu Chi Medical Foundation, Hualien 970, Taiwan; 15 Centre of General Education, Buddhist Tzu Chi Medical Foundation, Tzu Chi University of Science and Technology, Hualien 970, Taiwan; 16 Department of Medical Research, China Medical University Hospital, China Medical University, Taichung 404, Taiwan; 17 Proteomics Lab, National Centre for Cell Science, Pune 411007, India; 18 Thermo Fischer Scientific India Pvt Ltd, Whitefield, Bangalore 560066, India; 19 Department of Pharmacy Practice, Faculty of Pharmacy, Universitas Airlangga, Surabaya 60115, Indonesia; 20 School of Medical and Life Sciences, Sunway University, Bandar Sunway 47500, Malaysia; 21 Department of Pharmacy, Abdul Wali Khan University, Mardan 23200, Pakistan; 22 Zayed Center for Health Sciences, United Arab Emirates University, Al Ain 15551, United Arab Emirates; 23 Department of Biology, College of Arts and Sciences, Khalifa University, Abu Dhabi 127788, United Arab Emirates

**Keywords:** phytochemicals, curcumin, genistein, tanshinone, proteomics, diseases, therapeutic intervention

## Abstract

**Simple Summary:**

Over the years, alternative and complementary medicine have garnered much attention all across the globe. To this end, phytochemicals have intriguing potential against myriads of disease conditions; nevertheless, as of yet, the molecular intricacies for their therapeutic potential is incompletely understood. It is widely acknowledged that proteomics technology has been ex-plored as a reliable approach to understand the molecular intricacies related to phytochemi-cal-based therapeutic interventions. Reckoning with this, the present review provides an overview of the proteomics studies performed to unravel the underlying molecular intricacies of various phytochemicals such as Curcumin, Genistein, and Tanshinone IIA.

**Abstract:**

Phytochemicals possess various intriguing pharmacological properties against diverse pathological conditions. Extensive studies are on-going to understand the structural/functional properties of phytochemicals as well as the molecular mechanisms of their therapeutic function against various disease conditions. Phytochemicals such as curcumin (Cur), genistein (Gen), and tanshinone-IIA (Tan IIA) have multifaceted therapeutic potentials and various efforts are in progress to understand the molecular dynamics of their function with different tools and technologies. Cur is an active lipophilic polyphenol with pleiotropic function, and it has been shown to possess various intriguing properties including antioxidant, anti-inflammatory, anti-microbial, anticancer, and anti-genotoxic properties besides others beneficial properties. Similarly, Gen (an isoflavone) exhibits a wide range of vital functions including antioxidant, anti-inflammatory, pro-apoptotic, anti-proliferative, anti-angiogenic activities etc. In addition, Tan IIA, a lipophilic compound, possesses antioxidant, anti-angiogenic, anti-inflammatory, anticancer activities, and so on. Over the last few decades, the field of proteomics has garnered great momentum mainly attributed to the recent advancement in mass spectrometry (MS) techniques. It is envisaged that the proteomics technology has considerably contributed to the biomedical research endeavors lately. Interestingly, they have also been explored as a reliable approach to understand the molecular intricacies related to phytochemical-based therapeutic interventions. The present review provides an overview of the proteomics studies performed to unravel the underlying molecular intricacies of various phytochemicals such as Cur, Gen, and Tan IIA. This in-depth study will help the researchers in better understanding of the pharmacological potential of the phytochemicals at the proteomics level. Certainly, this review will be highly instrumental in catalyzing the translational shift from phytochemical-based biomedical research to clinical practice in the near future.

## 1. Introduction

**Figure 1:**
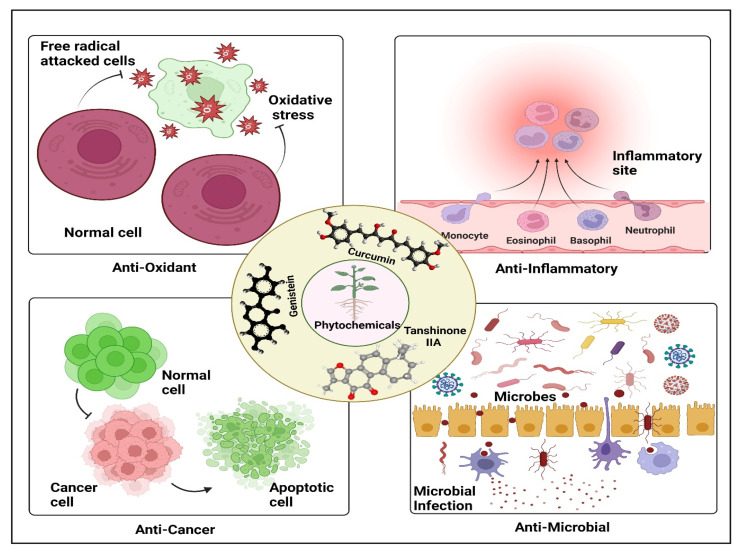
Representative figure highlighting some of the therapeutic potentials of curcumin, genistein, and tanshinone IIA. These phytochemicals have been shown to possess intriguing anti-microbial, anti-inflammatory, anti-oxidant, and anti-cancer potentials. The figures are prepared with the BioRender Software (biorender.com).

In recent years, alternative and/or complementary medicine have garnered much attention across the globe [1,2,3,4,5,6]. Phytochemicals are biologically active substances that embody various intriguing pharmacological properties against diverse pathological conditions including microbial infections, metabolic disorders, cancers, degenerative diseases, etc. The therapeutic potential of phytochemicals has been extensively investigated over the last few decades, and various reports have highlighted their interesting biological and therapeutic potentials [7,8,9,10,11]. Nevertheless, the molecular intricacies for their therapeutic potential are still an area of active research. Among various phytochemicals, curcumin (Cur), genistein (Gen), tanshinone IIA (Tan IIA), allicin, eugenol, apigenin, lycopene, anthocyanin, capsaicin, and shogaols share a history of high repute. Table 1 delineates the pharmacological properties, molecular functions, and therapeutic applications of various phytochemicals against diverse pathophysiological conditions. Of note, these phytochemicals embody diverse chemical space for drug discovery; to this end, various comprehensive online databases of phytochemicals have been developed, which enables computational approaches towards natural product-based drug discovery. These include TCM@Taiwan [12], KNAPSACK [13], TCMID [14], CVDHD [15], Nutrichem [16], TCM-Mesh [17], IMPPAT [18], etc. Interestingly, lately various newer therapeutic chemical moieties have been deciphered in pharmacology; interestingly, out of these identified therapeutic chemicals, approximately 50% are phytochemicals in nature, which depicts their importance in therapeutic interventions [19]. It is envisaged that dietary phytochemicals are extensively explored for therapeutic interventions due to a wide variety of reasons, including ease of availability, lower toxicity issues, characteristic biological effects, cost-effectiveness, and diversity of chemical components in plants. Several clinical studies have demonstrated a strong correlation between dietary intake of phytochemicals and reduced risk of cancer development and relapse [20,21,22,23,24]. A representative figure highlighting some of the therapeutic potentials of Cur, Gen, and Tan IIA are depicted in Figure 1.

### 1.1. Brief Overview on the General Characteristics of Various Phytochemicals

#### 1.1.1. Curcumin

Cur is an active lipophilic polyphenol compound with various beneficial biological activities. The therapeutic properties of Cur have long been reported for centuries [137,138]. Basically, Cur is a bioactive component of *Curcuma longa*, which belongs to the ginger family. Cur has high potential to scavenge reactive oxygen species (ROS) which makes it an important antioxidant and therapeutic molecule [139,140]. The properties of Cur extend to numerous beneficial functions such as anti-inflammatory, anti-microbial, anti-genotoxic, anti-neoplastic, anti-mutagenic, and anti-tumor activities [141,142,143,144,145]. Moreover, it has been shown to possess phototoxic and photodynamic potential as well [31,32,33]. Although it has low systemic bioavailability issues; nevertheless, interesting data exist to support its intriguing clinical evaluation [146].

#### 1.1.2. Genistein

Gen is an isoflavone mainly present in soy and soy-based food products that are consistently consumed by the Asian population [147,148]. Numerous epidemiological studies have indicated lower incidence of breast and prostate cancers in the Asian countries as compared to other Western countries. These observations have reinforced interest in focusing on the possible contribution of high dietary consumption of isoflavones and lower incidence of cancer [149,150]. It embodies striking structural similarity with the estrogen hormone as a result it is also known as phytoestrogen. Gen embodies a broad range of vital properties, such as antioxidant, anti-inflammatory, and anti-microbial; in addition, it is pro-apoptotic, anti-proliferative, and anti-angiogenic, which validates its chemo-preventive and chemo-therapeutic potential [45,151,152,153,154,155,156]. Interestingly, various clinical studies are on-going to ascertain its pharmacological potential, and there is great optimism that Gen formulation with better bioavailability could seemingly revolutionize Gen-based pharmacological interventions [42].

#### 1.1.3. Tanshinone IIA (Tan IIA)

Tan IIA is a major lipophilic component extracted from *Salvia miltiorrhiza* Bunge. Accumulating evidence has shown that Tan IIA exhibits multiple biological functions, such as anti-oxidative, anti-inflammatory, and anti-angiogenic effects, as well as anticancer activity against various types of cancers [157,158]. Studies have shown that Tan IIA significantly inhibits the proliferation of several types of tumors, blocks the cell cycle, and induces apoptosis and autophagic death in addition to inhibiting cell migration and invasion. However, poor bioavailability has been a major challenge for pharmaceutical development of Tan IIA, since Tan IIA is challenging to absorb directly in the intestine. Therefore, various analogs and/or formulations have been developed to overcome its bioavailability issues [11].

#### 1.1.4. Allicin

Allicin (diallylthiosulfonate) is obtained from *Allium sativum* (garlic). This sulfur compound provides a specific taste and smell to the freshly cut/crushed garlic. Allicin has displayed various intriguing pharmacological properties including potential anti-microbial agent. Accumulating evidence has shown that it embodies anti-bacterial activity against various Gram-positive and Gram-negative strains, and methicillin-resistant Staphylococcus aureus [64]. It also exhibits anti-fungal activity when used in in vitro and in vivo systems [159]. Studies have shown that allicin possess intriguing antioxidant potential; and the antioxidant potential of allicin could be plausibly ascribed to its ability to attenuate superoxide, nitric oxide (NO), and hydroxyl radicals [160]. Interestingly, it has been shown that the consumption of garlic in the diet was corelated with reduction of total cholesterol, low density lipoproteins, and triglycerides [161,162]. Moreover, it has been demonstrated to inhibit cholesterol biosynthesis seemingly owing to the inhibition of squalene-monooxygenase 85 and acetyl-CoA synthetase [68,69,163,164]. It has displayed intriguing anticancer potential against different types of cancers [70,71,72]. Moreover, it has been shown to exhibit an antihypertensive effect and neuroprotective potentials as well [66].

#### 1.1.5. Eugenol

It is a phenolic compound (phenylpropanoid) obtained from the leaves and buds of *Eugenia caryophyllata* (clove). It is chemically 4-allyl-2-methoxyphenol and it imparts the spicy aroma to cloves [165,166]. Several studies have demonstrated different bioactivities of eugenol including anti-bacterial, anti-fungal, anti-viral, antioxidant, and anti-inflammatory properties [167,168,169,170]. Moreover, it has exhibited intriguing potential to combat various types of cancers including gastric, colon, prostate, skin, breast cancer and other cancers [77,78,80,171] through various intricate mechanisms. In addition, it has been shown to embody antidiabetic [83], antiparasitic [84], antileishmanial [85], antipyretic [81], analgesic [172,173] anti-hypercholesterolemic, and antiatherogenic potentials as well [82].

#### 1.1.6. Apigenin

It is trihydroxyflavone found in chamomile, artichokes, celery, sorghum, parsley, oregano etc [174,175]. It embodies antioxidant properties [86] and it has been highlighted that the antioxidant potential of apigenin is plausibly mediated through modulation of antioxidant enzymes (catalase, superoxide dismutase, glutathione peroxidase, and phase II detoxification enzymes) and inhibition of the NF-κB signal transduction pathways [176,177]. Further, it is an intriguing anti-inflammatory agent [178], and the anti-inflammatory effects are seemingly mediated through inhibition of several cytokines including Th2 cytokines, IL-4, IL-10, NLRP3, interleukin 1β genes, etc besides other intricate mechanisms [89,179]. In addition, it also acts antibacterial [90], antiviral [91] and anticancer agent [180]. Similarly, the mechanism of anticancer potential is plausibly mediated through upregulation of STAT1 gene (tumor suppressor), and downregulation of IL-6, TNF-α, and CD40 (tumor causing genes) besides other intricate mechanisms [181]. Moreover, it exhibits antidiabetic activity [94] which has been seemingly attributed to various underlying mechanism including stimulation of insulin secretion, inhibition of gluconeogenesis, and increment in glycogen synthesis [94,95,96].

#### 1.1.7. Lycopene

It is a lipophilic carotenoid hydrocarbon compound found in orange, red, and pink coloured vegetables and fruits including tomatoes, melons, apricots, peaches, grapes, cranberries and papayas [182,183]. Lycopene exhibits various attractive potentials including antioxidant [184], anti-inflammatory [100], anticancer [185,186], cardioprotective [104], and neuroprotective potentials [105]. Studies have shown that lycopene displays intriguing antioxidant activity seemingly through enhancing the level of enzymatic antioxidants (catalase, peroxidase, and superoxide dismutase) as well as non-enzymatic antioxidants (vitamin C and E) [99,184]. Similarly, it displays anti-inflammatory activities plausibly through inhibition of several cytokines and chemokines including NF-κB, IL-6, IL-8, IL-1, TNF-α, nitric oxide (NO) etc. [187,188,189]. Lycopene acts as a cardioprotective agent seemingly through attenuation of oxidation of low density lipoproteins, increment of high density lipoproteinslevels besides other intricate mechanisms [190]. Likewise, the anticancer effect is attributed to induction of apoptosis, cell cycle arrest, and the amelioration of insulin-like growth factor 1 receptor (IGF-1R) signal transduction pathways and so-on [185,186,191].

#### 1.1.8. Anthocyanins

These are group of natural water-soluble phenolic compounds that are broadly distributed in several plant families including Rosaceae, Vitaceae, Cruciferae, Caprifoliaceae, Ericaceae, Saxifragaceae, and Fabaceae [192,193]. Basically, they are subgroup of flavonoids and are found in various plant parts, particularly fruits and flowers [192,194,195]. These have demonstrated several important bioactivities including antioxidant, anti-inflammatory, anticancer, anti-viral, attenuation of neurodegenerative diseases and prevention/treatment of cardiovascular diseases besides other intriguing pharmacological properties [196]. Interestingly, anthocyanins have been reported more potent antioxidants when compared to vitamins C and E [197]. Mechanistically, its antioxidant potential has been associated with its ability to modulate the antioxidant defense system, stimulate glutathione synthesis, and activate antioxidant enzymes (catalase, SOD, glutathione peroxidase). In addition, it has been shown to chelate several metal ions such as iron and copper, and henceforth, reduce free radical production through Fenton and other reactions [198]. Further, the anti-inflammatory effect of anthocyanins is mediated through multiple intricate mechanisms including inhibition of NF-kB, COX-2, TNF-α, IL-1β, and IL-6 [199,200]. Likewise, the anticancer potential of anthocyanins has been attributed to modulation of NF-kB, PI3K/Akt pathway resulting in reduced proliferation of tumor cells and induction of apoptotic responses [110,201].

#### 1.1.9. Capsaicin

It is an alkaloid found in the Capsicum genus. Capsaicin is lipophilic, colorless, odorless, crystalline capsaicinoid having the molecular formula C_18_H_27_NO_3_. Capsaicin exhibited various beneficial pharmacological properties. It has been shown to embody potent antioxidant activity; the antioxidant effect is seemingly attributed to modulation of antioxidant enzymes [202]. In addition, it has been shown to possess intriguing inflammatory potential and the anti-inflammatory potential of capsaicin has been attributed through inhibition of NF-*k*B, and cytokines levels [203]. Further, it has been highlighted to embody antidiabetic potential, which has been plausibly associated with its ability to induce improvement of glucose metabolism and glucose tolerance [204]. Moreover, capsaicin exhibits intriguing anticancer effect against different types of cancers [205,206]. The mechanism behind the anticancer activity is seemingly through induction of apoptotic responses, inhibition of angiogenesis and so-on [207]. It shows several other interesting pharmacological activities including gastroprotective [121], anti-obesity properties [122], and interestingly it is useful for chronic pain such as that occurring in diabetic neuropathy, rheumatoid arthritis, osteoarthritis, and musculoskeletal pain [208,209] with equal potency.

#### 1.1.10. Shogaols

Shogaols are phenylakylketones found in *Zingiber officinale*. It has been found as intriguing antioxidant [125] and evidence has shown that shogaols ameliorates oxidative stress seemingly through upregulation of phase II antioxidant enzymes such as heme oxygenase I, glutathione, and thioredoxin I, antioxidant response element promotor functions via the Nrf2 signaling pathway, and so on [126,127,210]. Moreover, it embodies anti-inflammatory, anti-emetic, and anti-thrombotic potential [136] and studies have highlighted that it attenuates inflammatory responses plausibly through multiple intricate pathways involving nuclear factor-kappa B, mitogen-activated protein kinase cascades, activator protein-1, and peroxisome proliferator-activated receptor gamma, etc. [129,130,131]. Furthermore, it exhibits anticancer potentials against various forms of cancers including breast, prostate, bowel, ovary, pancreatic cancer etc mainly through induction of apoptosis and cell cycle arrest [211].

## 2. Proteomics-Based Interventions in Phytochemical Studies

Proteomic analysis is an unbiased perspective, which is a convenient approach to get a global overview about the effectiveness of bioactive molecules [212,213,214]. Interestingly, proteomic studies could be the intriguing intervention in search of precise phyto-therapeutic agents to combat various diseases. Reckoning with these, in this review, we have majorly concentrated on the proteomic-based studies of Cur, Gen, and Tan IIA bioactive molecules. This review updates the most relevant informations by incorporating an extensive amount of proteomic-based research studies related to these bioactive molecules. A comprehensive table delineating the proteomic studies related to Cur, Gen, and Tan IIA is highlighted in Table 2.

**Figure 2:**
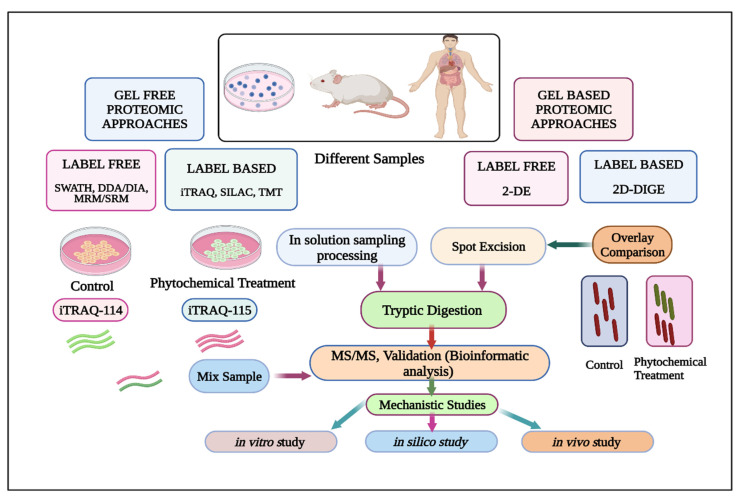
An overview of the proteomics strategies exploited to study the underlying intricacies for phytochemical-based therapeutic interventions. The figures are prepared with the BioRender Software (biorender.com). Abbreviations: SWATH: Sequential Windowed Acquisition of All Theoretical Fragment; DDA: Data-Independent Acquisition; DIA: Data-Dependent Acquisition; MRM: Multiple-Reaction Monitoring; SRM: Selective Reaction Monitoring; iTRAQ: Isobaric tag for Relative and Absolute Quantitation; SILAC: Stable Isotope Labeling by/with Amino acids in Cell culture; TMT: Tandem Mass Tag; 2-DE: Two-dimensional Electrophoresis; 2D-DIGE: Two-dimensional-Difference in Gel Electrophoresis; MS: Mass spectrometry.

Over the last two decades, various mass spectrometry (MS)-based high throughput proteomic approaches have been widely utilized for a plethora of applications [215,216]. As a matter of fact, the proteomic analysis deals with the profiling, identification, and quantification of proteins as well as peptides in different biological samples [217]. Conventionally, it comprises of various gel-based techniques such as two-dimensional gel electrophoresis (2-DE), two-dimensional differential gel electrophoresis (2D-DIGE), and gel free techniques. For example, these include stable isotope labeling by amino acids in cell culture (SILAC), isobaric tag for relative and absolute quantitation (iTRAQ), tandem mass tags (TMT), label free quantitation (LFQ) analysis, and multiple reaction monitoring (MRM). For a comprehensive review on proteomics-based technology, please refer to articles by Beck et al., [218] and Domon et al., [219]. Here, we have described the proteomics-based studies of Cur, Gen, and Tan IIA in detail. An overview of the proteomics strategies exploited to study the underlying molecular intricacies for phytochemical-based therapeutic interventions has been depicted in Figure 2.

**Table 2:** Representative table providing an overview of various proteomics studies to understand the potential role of Curcumin, Genistein, and Tanshinone IIA against various disease pathologies.

SI. No.	Target Tissue or Cells	Proteomic Strategies	Objective of the Study	Prospective Proteins Upregulated (↑) or Downregulated (↓)	Disease and/or Condition Studied	References
Proteomics studies to understand the molecular intricacies of Cur						
1	Bacillus subtilis AH75 strain	2D-DIGE, iTRAQ	To investigate the proteome alterations in Bacillus subtilis following Cur treatment and identification of its molecular/cellular targets to understand the mechanism of action	UDP-N-acetyl glucosamine 1-carboxy vinyl transferase 2—↓ Putative septation protein (SpoVG)—↑ ATP-dependent zinc metalloprotease (FtsH)—↑	Antibacterial action	[142]
2	Escherichia coli (ATCC 25922)	LFQ, LC-MS/MS	To investigate the mechanistic aspects of the antibacterial effects of Cur in the dark and upon illumination	Chaperone SeqB—↑ Gro-P like protein E (GrpE)—↑ Elongation factor (Tu1)—↑ Universal stress *protein* F (UspF)—↑ Probable quinol monooxygenase (YgiN)—↑ Uncharacterized oxidoreductase (YajO)—↑	Antibacterial action	[220]
3	Imipenem- resistant Acinetobacter baumannii	LFQ, LC-MS/MS	To investigate the alteration in protein profile following exposure to blue light combined with Cur treatment	Carbonylated Omp38—↑ Carbonylated elongation factor Tu and P—↑ Carbonylated ribosome releasing factor—↑	Antimicrobial resistance	[221]
4	Fathead minnow epithelial cells (FHM)	LFQ, LC-MS/MS	To evaluate the effect of Cur pretreatment in fathead minnow cells infected with viral hemorrhagic septicemia virus (VHSV)	Fibronectin (FN) 1—↓ Heat shock cognate 71 (HSC71)—↓ F-actin—↑	Viral Hemorrhagic Septicemia	[29]
5	Human liver carcinoma cells (HepG2 cells)	2D-DIGE, MALDI-TOF/ TOF/MS	To understand the anticancer mechanism of natural borneol (NB) and Cur in combination	Heterogeneous nuclear ribonucleoprotein (hnRNPC1/C2)—↓ Nucleophosmin (NPM)—↓ Proteasome 20S Subunit Alpha 5 (PSMA5)—↓	Liver cancer	[222]
6	Human glioblastoma *cells* (U87 cells)	2D-DIGE, iTRAQ, LFQ LC-MS/MS	To understand the underlying intricacies of LLL12, a Cur derivative against glioblastoma multiforme	Triose phosphate isomerase (TPI)—↓ Phosphoglycerate mutase 1 (PGAM1)—↓ Adaptor molecule (CRK2)—↓ protein DJ-1 (PARK7) —↓ Basic transcription factor 3 (BTF3)—↓	Glioblastoma multiforme	[223]
7	Human colorectal carcinoma cells (HCT116 cells)	iTRAQ (TM)	To understand the molecular mechanism of action of Cur against colon cancer and try to identify its exact molecular targets	Microtubule-associated proteins 1A/1B light chain 3B (LC3B)—↑ Lysosomal-associated membrane protein (Lamp1)—↑ Heat shock protein 70 (HSP70)—↑	Colorectal cancer	[224]
8	Melanoma cells (LB24 Dagi cells)	LFQ LC-MS/MS	To investigate the changes in the protein profile of melanoma cells following treatment with D6 (Cur analog)	PolyUbiquitin-C—↑ Heat shock 70 kDa protein 1A/1B—↑ DnaJ homolog subfamily B member 1—↑ Heterogeneous nuclear ribonucleoprotein Q—↓ Histone-H2A type 1-C—↓	Melanoma cancer	[225]
9	Human liver carcinoma cells (HepG2 cells)	MALDI- TOF/TOF/ MS	To understand the efficacy of Cur/β-cyclodextrin polymer (CUR/CDP) inclusion complex against HepG2 and its possible molecular mechanisms of action	Nucleophosmin (NPM1)—↓ Peroxiredoxin-6 (PRDX6)—↓	Liver cancer	[226]
10	Human acute lymphocytic leukemia cells (MOLT-4 cells)	2-DE, MALDI-TOF Pro	To understand the role of Siah- interacting protein (SIP) in Cur-based therapeutic intervention	Siah-interacting protein (SIP)—↓	Leukemia	[227]
11	Human lung adenocarcinoma cells (A549 cells)	2-DE, MALDI-TOF/ TOF MS	To understand the precise molecular mechanism of Cur against human lung cancer	Heat shock protein 90 (HSP-90)—↓ 14-3-3 protein—↓	Lung cancer	[228]
12	Human colon adenocarcinoma cells (LOVO cells)	MALDI- TOF/TOF MS	To investigate the action of irinotecan and Cur against colorectal cancer (LOVO) cells	Peroxiredoxin-4—↑ Glutathione S-transferase Mu 5—↓ Translocon associated protein subunit delta—↓ Calpain small subunit 1—↓ Protein disulfide-isomerase—↑ (Cur + irinotecan treatment)	Colorectal cancer	[229]
13	Human colorectal cancer cells (SW480 and SW620 cells)	LFQ LC-MS/MS	To understand the anti-metastatic properties of the conventional chemotherapeutic drugs and the phytochemicals through comparative proteomic approach	Fatty acid synthase (FASN)—↓ Histone H4—↓	Colorectal cancer	[230]
14	Human colon carcinoma cells (HCT-8/VCR cells)	2-DE, MALDI- TOF/ MS	To explore the differential proteomic profile of vincristine-resistant HCT-8/VCR cells with and without Cur treatment	Glutathione S-transferase pi1 gene (GSTP1)—↓	Colorectal cancer	[231]
15	Human colorectal cancer cells (SW480 and SW620 cells)	2-DE, LC-MS/MS	To understand anticancer activity of Cur against colorectal cancer	Mitogen-activated *protein* kinase (MEK1/2)—↑ Extracellular signal-regulated kinases (ERK1)—↑ Histone deacetylase C1 (HDAC1)—↓ Tumor protein 53 (P53)—↓ AMP-activated *protein* kinase (AMPKβ1)—↓	Colorectal cancer	[232]
16	Human gastric cancer cells (BGC-823, MKN-45 and SCG-7901 cells)	2-DE, MALDI-TOF/ TOF MS	To investigate the potential of Cur as natural anticancer agent against gastric cancer	Annexin A1 (ANXA1)—↑ Apoptosis Inducing Factor Mitochondria Associated 1 protein (AIFM1)—↑ Proliferation associated protein (A2G4)—↑ Protein phosphatase PP1-alpha catalytic subunit (PP1A)—↑ Glucose-regulated protein 75 (GRP75)—↓ T-complex protein 1 subunit alpha isoform a (TCPA)—↓ Eukaryotic initiation factor 4A-III (IF4A3)—↓ Thioredoxin domain-containing protein 5 (TXND5)—↓	Gastric cancer	[233,234]
17	Human breast cancer cells (MCF-7, ZR-75-1) and TGF-β1 pretreated fibroblasts	LFQ, LC-MS/MS	To investigate the effects of Cur against breast cancer	Heme Oxygenase-1 (HMOX1)—↑ Ras Related GTP Binding A (RRAGA)—↑ Ring Finger And CCCH-Type Domains 1 (RC3H1)—↓ (in MCF-7/CLC co-culture) Retrotransposon-derived protein (PEG10)—↓ (in ZR-75-1/CLC co-culture)	Breast cancer	[235]
18	Triple negative breast cancer (TNBC) cells (MDA-MB-231)	LFQ, LC-MS/MS	To gain insights into the molecular intricacies of the anticancer effects of combinatorial treatment of Cur and electrical pulses (Cur+EP) compared to solitary treatments	Aldolase, Fructose-Bisphosphate A (ALDOA)—↓ Enolase 2 (ENO2)—↓ Lactate dehydrogenase A (LDHA)—↓ Lactate dehydrogenase B (LDHB)—↓ Phosphofructokinase platelet (PFKP)—↓ Phosphoglucomutase 1 (PGM1)—↓ (PGAM1)—↓ Phosphoglycerate kinase 1 (PGK1)—↓	Triple negative breast cancer	[236]
19	Human oral adenosquamous carcinoma cells (CAL 27 cells)	SILAC, LC-MS/MS	To investigate the underlying molecular intricacies of tyrosine signaling in response to Cur	Tyrosine-protein phosphatase non- receptor type 6 (PTPN6)—↑ Abelson tyrosine-protein kinase 2 (ABL2)—↑ Fyn-related Src family tyrosine kinase (FRK)—↓ Pseudopodium enriched atypical kinase (PEAK1)—↓	Head and Neck cancer	[237]
20	Human Chronic myelogenous leukemia (CML) cells (K562 and LAMA84 cells)	SWATH MS	To investigate that how exosome proteins from Cur-treated K562 cells can mediate the anti-angiogenic effect on HUVECs	Myristoylated Alanine Rich C- Kinase Substrate (MARCKS)—↓ Ras Homolog Family Member B (RhoB)—↓ Vascular cell adhesion protein 1 (VCAM1)—↓	Chronic myelogenous leukemia	[238]
21	Human chronic myelogenous leukemia (CML) cells (K562 and LAMA84 cells)	LFQ, LC-MS/MS	To understand the pharmacological potential of Cur as a safe anti-tumor agent that can function as a chemosensitizer and a multi-targeted inhibitor	Aldolase, Fructose-Bisphosphate A (ALDOA)—↓ pyruvate kinase muscle isozyme (PKM)—↓ Lactate dehydrogenase A (LDHA)—↓ Phosphoglycerate kinase 1 (PGK1)—↓ Importin-7 (IPO7)—↓	Chronic myelogenous leukemia	[239]
22	Mouse macrophage cells (RAW264.7 cells)	2-DE, MALDI-TOF/MS	To study the anti-atherosclerosis mechanism of action of Cur	ATP synthesis H+ transporting—↑ MHC class II—↑ Non-muscle myosin alkali light chain—↑ Cytochrome b5—↑ Phosphodiesterase 4D—↓ Eukaryotic initiation factor 3 (elF-3)—↓ Hnrpf protein—↓ Vimentin (VIME)—↓ Nucleophosmin—↓ Ran binding protein (Ranbp 1)—↓	Atherosclerosis	[240]
23	Mouse fibroblast cells (3T3-L1) and Primary white adipocytes	2-DE, MALDI-TOF/MS	To understand the proteomic changes in cultured white adipocytes in response to Cur treatment and to identify the target proteins responsible for the fat-browning effects of Cur	Hormone-sensitive lipase (HSL)—↑	Weight management	[241]
24	C57BL/6 mice	LFQ, LC-MS/MS	To understand the therapeutic efficacy of Cur against pulmonary fibrosis	Tumor protein (p53)—↓ Urokinase-type Plasminogen Activator (Upa)—↑ Plasminogen activator inhibitor-1 (PAI-I protein)—↓	Pulmonary fibrosis	[242]
25	Human neuroblastoma (NB) cells (SH-SY5Y cells)	LFQ, LC-MS/MS	To explore the anticancer activity of Cur against human neuroblastoma	Heat shock protein 70 (Hsp70)—↑ Peroxiredoxin 1 (PRDX1)—↓ Peroxiredoxin 6 (PRDX6)—↓	Neuroblastoma	[243]
26	Human lens epithelial B3 cells (HLE- B3 cells)	SELDI-TOF/MS	To understand the effect of Cur on HLE-B3 cell proliferation	Chemotactic factor A17—↓ Chemotactic factor A22—↓ IL-8—↓ Neutrophil active peptide-2—↓	Posterior capsular opacification post cataract complications	[244]
27	Human colorectal adenoma	LC-MS/MS	To evaluate the effect of Cur on intestinal Uridine diphosphate glucuronosy ltransferase (UGT) expression	Uridine diphosphate glucuronosyltransferase (UGTs)—not affected by oral Cur	Colorectal adenoma	[245]
28	Hepatic liver tissue and Murine hepatocyte cells (AML12 cells)	2-DE, MALDI–TOF/MS	To investigate the underlying intricacies of the effect of Cur against non-alcoholic fatty liver disease (NAFLD)	Superoxide dismutase 1 (SOD1)—↑ Sirtuin 1 (SIRT1)—↑	Non-alcoholic fatty liver disease	[246]
29	Male hamsters	LFQ, LC-MS/MS	To elucidate the potential use of Cur and to identify its novel molecular targets	S100A6—↓ Lumican—↓ Plastin-2—↓ 14-3-3 zeta/delta—↓ Vimentin (VIME)—↓	Cholangiocarcinoma	[247]
30	Sprague Dawley rats	2-DE, LC-MS/MS	To investigate whether Cur regulates γ- enolase expression in focal cerebral ischemic injury in rats	γ-enolase—attenuation of its decreased expression induced by ischemic injury	Cerebral Ischemia	[248]
31	Adult male rats	2-DE; LC-MS/MS	To identify various proteins that are differentially expressed by Cur treatment in focal cerebral ischemia	Ubiquitin carboxy-terminal hydrolase L1 (UCH-L1)—↑ Isocitrate dehydrogenase (ICDH)—↑ Adenosyl homocysteinase (AHC)— ↑ Eukaryotic initiation factor 4A (eIF4A)— ↑ Pyridoxal phosphate phosphatase (PPP)—↓	Cerebral Ischemia	[249]
32	Mouse models of human inflammtory bowel disease (IBD)	2-DE, LC-MS/MS	To understand the role of nutrient–gene interactions in human inflammatory bowel disease (IBD)	Proteins involved in digestion, excretion, and metabolism—↓ Cellular stress and immune response proteins—↑	Inflammatory bowel disease	[250]
Proteomics studies to understand the molecular intricacies of Gen						
33	Triple negative breast cancer (TNBC) cells (MDA- MB-231 cells)	TMT	To elucidate anticancer effects of Gen against TNBC cells	Cyclin-dependent kinase inhibitor (p21WAF1)—↑ Bcl-2-associated X protein (Bax)—↑ B-cell lymphoma-2 (Bcl-2)—↓ Tumor p53 protein (p53)—↓ Cyclin-dependent kinase 1 (CDK1)—↓	Breast cancer	[251]
34	Human breast cancer cells (T47D cells)	SILAC	To understand the effect of a varying intracellular ERα/ERβ ratio on Gen-induced genes and protein expression profile	Myosin (MYH10, MYH14, MYL12B, MYH9, and MYL6)—↑ S100 family Ca2+ binding proteins (S100A8, S100A9)—↓ Prolactin Induced *Protein* (PIP)—↓	Breast cancer	[252]
35	Human hepatocellular carcinoma cells (SNU-449 cells)	2-DE, LC-MS/MS	To understand the anticancer effects of Gen against SNU-449 cells	B-cell lymphoma-2 (Bcl-2)—↓ Thioredoxin 1 (Trx1)—↓	Hepatocellular carcinoma	[253]
36	Human gastric cancer cells (SGC-7901 cells)	SDS-PAGE, LC- MS/MS	To understand Gen-induced protein alterations in gastric cancer cells and to investigate the molecular mechanism responsible for the anticancer actions	Kinesin family proteins (KIFs)—↓ KIF11, KIF20A, KIF22, KIF23—↓ Centromere *Protein* F (CENPF)—↓	Gastric cancer	[254]
37	Human promyelocytic leukemia cells (HL-60 cells)	In-gel digestion, MALDI-TOF/TOF/MS	To elucidate the changes in protein profile following treatment with Gen in human leukemia cells	Hsp70 protein 8—↑ Heterogeneous nuclear ribonucleoprotein (hnRNP) H1—↑ Ras-related protein (Rab14)—↓ Heterogeneous nuclear ribonucleo protein C (hnRNP C)—↓ Stathmin-1—↓	Leukemia	[17]
38	Human promyelocytic leukemia cells (HL-60 cells) and Human AML cells (MV4-11 cells)	iTRAQ	To investigate the prospect of using Gen as an effective alternative therapy for AML	Eukaryotic translation initiation factor 4E-binding protein 1 (4EBP-1)—↓	Acute myeloid leukemia	[255]
39	Mouse Cardiomyocyte cells (HL-1 cells)	2-DE, LC-MS/MS	To study the concentration-dependent effects of Gen treatments on cardiomyocytes	Heat shock protein 27 (HSP27)—↑ Cathepsin D—↑ Heat shock protein (HSP70)—↑ Glucose-regulated protein (GRP78)—↑ Voltage-dependent anion-selective channel protein 1 (VDAC-2)—↑ Preprotein translocase of the inner membrane of mitochondria (TIM50)—↑ Bcl-2-associated athanogene 2 (BAG2)—↓ N-Myc Downstream Regulated 1 (NDRG1)—↓	Cardiac Ischemic stress	[256]
40	Human endothelial cells (EA. hy 926 cells)	2-DE, LC-MS/MS	Gen, on changes in protein expression levels induced by the endothelial stressor homocysteine (Hcy) in EA.hy 926 endothelial cells	End binding 1 (EB1)—↓ Cathepsin B—↓	Atherosclerosis	[257]
41	Rat Hepatic Stellate cells (HSC-T6 cells)	2-DE, LC-MS/MS	To elucidate the antifibrotic mechanism of combinatorial treatment of Gen, Taurine, and epigallocatechin gallate (EGCG)	Hexokinase 2 (HK2)—↓ Lysosomal-associated membrane protein (LAMP1)—↑ Cathepsin D—↑	Liver fibrosis	[258]
42	Rats	2-DE, LC-MS/MS and/or MALDI-TOF/TOF	To understand the role of Gen in breast cancer prevention	Endoplasmic reticulum resident protein 29 (ERp29)—↑ Guanine deaminase—↑ Fetuin-B—↑ Annexin A1, A2—↓	Breast cancer	[259]
43	Rats	2-DE, LC-MS/MS	To investigate Gen mechanisms of action against chemically induced mammary cancer	GTP cyclohydrolase 1 (GTP-CH1)—↑ Tyrosine hydroxylase (TH)—↑ Vascular endothelial growth factor receptor 2 (VEGFR2)—↓	Breast cancer	[260]
44	Prepubertal girls blood and urine	TMT	To identify protein biomarkers of the effect and susceptibility for cancer from the blood of girls exposed to select environmental chemicals	Endothelin-converting enzyme (ECE-1)—↓ Eukaryotic translation initiation factor 3 subunit J (EIF-3)—↓ Nucleolar 7—↑ PR domain zinc finger 5 (PRDM5)—↑	Mammary cancer	[261]
45	Sprague Dawley rats	TMT	To identify protein biomarkers of susceptibility from blood sera of rats exposed prepubertally to Bisphenol A (BPA) or Gen	Neurosecretory protein VGF 8a (VGF)—↓ Rho-associated coiled-coil containing protein kinase 2 (ROCK2)—↓ Matrix metalloproteinase 3 (MMP3)—↓ Protein tyrosine phosphatase receptor type K (PTPRK)—↑ SET domain containing 2 (SETD2)—↑ Ubiquitin carboxyl-terminal hydrolase—↑	Cancer	[262]
46	C57BL/6J female mice (INTACT) and castrated females (CAST)	2-DE, LC-MS/MS	To assess the impact of Gen on the cardiac proteome in ovariectomized female mice	Myosin 6—↑ Myosin regulatory light chain 4 (MLC-4)—↑ Moesin—↑	Cardiovascular disease	[263]
47	Mice	2-DE, LC-MS/MS	To characterize an animal model for alternative hormone replacement with Gen as a natural estrogenic compound	LIM domain-binding protein—↑ Desmin—↑	Animal model characterization	[264]
48	Sprague Dawley rat model of liver fibrosis	iTRAQ	To understand the anti-fibrotic mechanisms of combination therapy of Gen, taurine, and epigallocatechin gallate (EGCG)	Annexin A2 (Anxa2)—↑ Thioredoxin domain-containing protein (Txn1)—↑ Proteoglycan 4 (Prg4)—↑ polymeric immunoglobulin receptor (Pigr)—↑ fibulin-1 (Fbln1)—↑ Triosephosphate isomerase (Tpi1)—↑	Liver fibrosis	[265]
49	Rat model of liver fibrosis	iTRAQ	To understand the possible therapeutic mechanism of combination therapy against liver fibrosis	Thioredoxin domain-containing protein (Txn1)—↑ DEAD box protein family (Ddx39a)—↑ 17-beta-hydroxysteroid dehydrogenase type 6 (Hsd17b6)—↑ Cysteine Conjugate-beta Lyase 2 (Ccbl2)—↑ Magnesium cation transporter protein (Magt1)—↑ Cytochrome P450 4A14 (Cyp4a14)—↑ Glutathione S-transferase A1 (Gsta1)—↑	Liver fibrosis	[266]
50	Adults male Wistar rats	SDS-PAGE, LC- MS/MS	Morphological response of reactive astrocytes positive for glial fibrillary acidic protein (GFAP) in rats	Glial fibrillary acidic protein (GFAP)—↓	Astrogliosis	[267]
Proteomics studies to understand the molecular intricacies of Tan IIA						
51	Human cervical cancer cells (HeLa cells)	In-gel digestion, MALDI-TOF MS	To investigate the prospective potential of Tan IIA as a potential anti-tumor agent	Proliferating cell nuclear antigen (PCNA)—↓ Heat shock protein 27 (HSP27)—↓ Vimentin (VIME)—↓ β-tubulin—↑ Superoxide dismutase (MgSOD)—↑ Glucose-regulated *protein 75* (GRP75)—↑ Prohibitin—↑	Cervical cancer	[268]
52	Human hepatocellular carcinoma cells (MHCC97-H cells) and Chang liver cells	LFQ, LC-MS/MS	To elucidate the Tan IIA-induced protein profile alteration in MHCC97-H cells	Keratin, type II cytoskeletal 8—↓ Keratin, type I cytoskeletal 18, 19, and 20—↓ Cathespin D—↓ Profilin 1—↓ Nucleoside diphosphate kinase A—↑ Annexin A1, A2—↑	Liver cancer	[269]
53	Human gastric cancer cells (AGS cells)	iTRAQ	To understand the mechanism of action of TanIIA against gastric cancer	Tumor p53 protein (P53)—↑ Serine/threonine-*protein* kinase (AKT)—↓	Gastric cancer	[270]
54	Immortalized rat myofibroblast cells (HSC-T6 cells) and Human Hepatocellular carcinoma cells (HepG2 cells)	In-gel digestion, MALDI-TOF/ TOF/ MS	To explore the mechanism of apoptosis induced by Tan IIA on activated rat Hepatic Stellate Cells (HSCs)	Prohibitin—↑ Translational Controlled Tumor Protein–(TCTP)—↓ GDP-dissociation inhibitor 1 (GDIR1)—↓ 14-3-3ε—↓	Liver fibrosis	[271]
55	Osteosarcoma cancer cells (MG-63 cells)	2-DE, LC-MS/MS	To understand the molecular mechanisms of anticancer effects of Ginsenoside Rg1, Cinnamic acid, and Tan IIA and to know their targets	Prohibitin—↓	Osteosarcoma	[272]
56	Human gastric cancer cells (AGS cells)	Phosphoproteomics LTQ LC-MS/MS	To understand the molecular signal transduction pathway associated with the anticancer potential of Tan IIA	Phosphorylation of heat shock protein 27 Heat shock factor 1 (HSF1)—↑	Gastric cancer	[273]
57	Human papillomavirus type 16 (HPV-16)-positive cells (CaSki cells) and Human cervical cancer cells (HeLa and SiHa cells)	In-gel digestion, MALDI-TOF/ TOF MS	To evaluate the growth inhibitory effect of Tan IIA on CaSki cells	Protein disulfide-isomerase A1 (PDIA1)—↓ Glucose-regulated protein 78 (Grp78)—↓ Whey proteins (TERA)—↓ Glucose-regulated protein (Grp94)—↓ Vimentin (VIME)—↓ Glucosidase II Alpha Subunit (GANAB)—↑ Cytoskeletal protein (VINC)—↑ The Putative Coupling Protein (TCPA)—↑ The brucella effector protein B (TCPB)—↑ Keratin 2C7 (K2C7)—↑	Cervical cancer	[274]
58	Bone marrow- derived mesenchymal cells (BM-MSC) and Wharton’s Jelly- derived mesenchymal cells (WJ- MSC)	LFQ, LC-MS/MS	To understand the ability of BM- and WJ-MSC to differentiate towards the osteogenic lineage	Proteins of BMP signaling—↑	Orthopedic disease	[275]
59	Mice myocardial cells	LFQ, MALDI- TOF/TOF/MS	To investigate the effect of Tan IIA on transverse aortic constriction (TAC)-induced heart failure	NADPH Oxidase 4 (Nox4)—↓ P38—↓ Nuclear factor erythroid 2- Related factor 2 (Nrf2)—↑	Myocardial apoptosis	[276]
60	Lung cancer Radioresistant cells (H358-IR and H157-IR cells)	SILAC MS	To evaluate the potential of Tan I as a potential radiation sensitizer in lung cancer	Phosphoribosyl Pyrophosphate Amidotransferase (PPAT)—↓ B-cell lymphoma-2 (BCL2)—↓ Caspase 8—↑	Lung cancer	[277]
61	Adult male KM mice	2-DE, LC-MS/MS	To understand the potentials of Tan II A sodium sulfonate (TSNIIA-SS) against Doxorubicin (DXR)-induced nephropathy	Myo-inositol oxygenase—↑ Glutathione peroxidases (GSH-Pxs)—↓ Proteasome alpha 5—↓	Nephropathy	[278]

Abbreviations: LFQ: Label free quantification; MALDI-TOF/TOF/MS: Matrix Assisted Laser Desorption/Ionization-Time of Flight/Time of Flight/Mass Spectrometry; 2-DE: Two-dimensional Electrophoresis; iTRAQ: Isobaric tag for relative and absolute quantitation; LC-MS/MS: Liquid Chromatography-Mass Spectrometry/Mass Spectrometry; 2D-DIGE: Two-dimensional-Difference In Gel Electrophoresis; SILAC: Stable Isotope Labeling by/with Amino acids in Cell culture; SELDI-TOF-MS: Time of Flight Mass Spectrometry; SDS-PAGE: Sodium dodecyl-sulfate polyacrylamide gel electrophoresis; TMT: Tandem Mass Tag.

### 2.1. Proteomic Approaches to Understand the Function of Curcumin as Therapeutic Intervention

#### 2.1.1. Proteomic Studies to Explore Its Potential against Microbial Diseases

Accumulating evidence has highlighted that Cur has a broad spectrum of anti-microbial effects including antibacterial, antifungal, and antiviral action [29,142,220,221]; it has been envisaged to exhibit synergistic effects in combinatorial therapeutic regimes. In this regard, to explore the antibacterial activities of Cur against *Bacillus subtilis*, Reddy et al. treated *B. subtilis* AH75 strain with Cur (20 μM) at different time intervals, and performed a comprehensive proteomic analysis using 2D-DIGE and iTRAQ to analyze the differential expression profile. Interestingly, differential proteomics profiling revealed alterations in various proteins including putative septation protein SpoVG, UDP-N-acetylglucosamine 1-carboxy vinyl transferase 1, and the ATP-dependent Clp protease proteolytic subunit. Moreover, alteration in the universal chaperone system (GroEL), required for the tubulin homologue protein filamenting temperature-sensitive mutant Z (FtsZ) folding, as well as the major protease (Clp family) system that targets FtsZ for degradation was also observed. Further, bioinformatics analysis revealed that Cur treatment considerably altered various cellular processes including central metabolism, fatty acid metabolism, and cell wall synthesis pathways, all of which have an important role for bacterial viability. Collectively, the study provided a plausible understanding of the mechanism of action and the putative targets of Cur, suggesting that treatment of Cur majorly affects cell division, cell wall synthesis, chaperones, and central metabolism in *B*. *subtilis* AH75 strain [142].

Reckoning with its antiviral action, it has been envisaged that although Cur has been widely studied in the background of the antiviral mechanism, the available literature does not clearly explain the effect of Cur in the early stages of viral infection. However, the underlying intricacies employed for interactions among viruses, cells, and antiviral compounds are incredibly diversified. Thus, it is important to comprehensively analyze the diverse protein–protein interactions in host cells during the viral entry phase. To this end, Jeong and colleagues investigated the underlying intricacies; for this, they pre-treated the head minnow cells with Cur (15–240 μM) followed by a viral hemorrhagic septicemia virus (VHSV) infection [29]. Thereafter, they performed a comparative proteomic study on the animal models with VHSV-infected, and Cur-treated VHSV-infected animals. Proteomics analysis revealed alterations in protein expression of several proteins including heat shock cognate 71 (HSC71), elongation factor 1 (EEF1), alpha cardiac muscle (ACTC1) protein and actin protein. Further pathway analysis through ingenuity pathways analysis (IPA) provided clues that HSC71 could be the primary candidate interacting with actin proteins (ACTB, ACTG, and F-actin), fibronectin (FN)-1, and gelsolin (GSN) in both VHSV-infected and Cur-treated VHSV-infected organisms. All these data provided evidence that Cur downregulates the expression of HSC71, which consequently increased virally infected cell viability, and inhibited the VHSV replication. Furthermore, Cur induced an alteration in the ratio of F-actin/G-actin, this represents another interesting connecting link that indicates the plausible mechanism to inhibit viral entry [29].

Collectively, all these studies provided a plausible understanding of the mechanism of action and the putative targets of Cur for their antimicrobial potential.

#### 2.1.2. Proteomic Studies to Explore Its Potential against Cancer

Several promising studies have shown the protective potential of Cur against many disease conditions; nevertheless, their anticancer potential is the most researched topic. Cur has been shown to inhibit cancer cell growth, invasion, and the metastasis properties of various types of cancer [233,279]. Further exploration of the underlying intricacies highlighted that the target proteins of Cur were found to be involved in many different processes including cell proliferation, apoptotic responses, nucleic acid processing, protein folding, protein translational machinery, proteolysis process, cytoskeleton organization, and signal transduction pathways [233].

Cur is well known for its therapeutic activities; nevertheless, it has poor systemic bioavailability. Therefore, various chemical analogs and/or Cur formulations have been developed with the aim to improve their bioavailability issues and enhance its efficacy thereof [280]. To this end, in order to increase the absorption of Cur, Natural borneol (NB), the bicyclic organic compound, has been formulated and tested against liver cancer. Interestingly, the treatment of Cur/NB in HepG2 cells resulted in differentially expressed proteins (17 proteins upregulated and 12 downregulated) that were functionally associated with the cell cycle and apoptosis, as well as the p53 pathway (hnRNPC1/C2, NPM, and PSMA5). To this end, decreased levels of hnRNPC1/C2 and NPM eventually leads to phosphorylation of the p53 protein; the activated p53, along with differentially expressed PSMA5, consequently increased the level of p21. Moreover, NB/Cur also enhances ROS synthesis, which is involved in the G2/M cell arrest mechanism. Collectively, it is reasonable that this proteomic study provided a strong evidence and better understanding regarding the anticancer property of Cur [222].

Further, Cur derivatized inhibitory compound, LLL12, demonstrated intriguing potential against glioblastoma multiforme (GBM) [223]. LLL12 is a known inhibitor of signal transducer and activator of transcription 3 (STAT3), which is constitutively active in various types of cancers. Basically, the global effects of targeting STAT3 using LLL12 were identified using 2D-DIGE and iTRAQ, suggesting intriguing anti-tumorigenic activity of LLL12. Interestingly, LLL12 treatment exhibited downregulation of phosphoglycerate mutase 1 (PGAM1), triosephosphate isomerase (TPI), adaptor molecule cysteine-rich receptor-like protein kinase 2 (CRK2), basic transcription factor 3 (BTF3), and protein DJ-1 (PARK7), which suggested that these targets may serve as prognostic or predictive markers in GBM [223]. Concomitantly, this study revealed Cur involvement in various cellular responses such as apoptosis induction, cellular metabolism, and anti-angiogenic activities.

Further, to better understand the molecular target of Cur, an intriguing investigation was performed by Wang and group in 2015. In their study, they utilized a cell-permeable Cur probe (Cur-P) coupled with an alkyne moiety that can be tagged with biotin for further enrichment. The researchers performed a quantitative proteomics approach to identify specific binding targets. This study revealed 197 proteins that were seemingly identified as Cur-binding targets. Further investigation divulged the target distribution and enrichment in different organelles such as mitochondria, nucleus, and plasma membrane. IPA divulged the anticancer effects of Cur, which suggest the involvement of Cur in a myriad of biological functions including mTOR signaling, mitochondrial dysfunction pathways, as well as regulation of eIF4/p70S6K and EIF2 proteins. Later, functional validation established that Cur induces autophagy, suppresses cellular protein synthesis, and increases ROS production and lysosomal activation, which leads to cell death of cancerous cells and confirms its anticancer potential [224].

Another investigation highlighting the antitumor activity of Cur D6 (hydroxylated biphenyl compound) on primary melanoma LB24Dagi cells, employed proteomics and mass spectrometry analysis. The altered proteins exhibited strong activation of a cellular stress response, with upregulation of several HSPs and triggered ubiquitin-proteasome pathways. The researchers concluded that Cur seemingly altered the majority of cellular functions and finally drives the cells to apoptotic pathways, without affecting normal healthy cells [225].

Another interesting study utilizing a gel-based proteomic approach showed decreased spot intensity (up to 70–90%) of SIP (Siah-interacting protein) in the Cur-treated (Cur sensitive) compared to the Cur-resistant human acute lymphocytic leukemia (MOLT-4) cells that caught the attention of the researchers. The study indicated that seemingly SIP is an important player in Cur-induced apoptosis in Cur sensitive cells and plays a critical role in Cur resistance [227].

Further, treatment of hypotriploid human epithelial lung carcinoma cells, viz., A549 cells with the Cur analog T63 (4-arylidene), revealed ~66 proteins with altered expression patterns. It seems that T63 contains a diverse range of molecular targets including HSP90 and 14-3-3 proteins as revealed by 2-DE and Ultraflex II MALDI-TOF/TOF MS analysis. Overall, the study proposed that T63-triggered cell cycle arrest and apoptotic responses involving mitochondrial dysfunction and ROS generation; and inhibition of the proteasomal machinery [228].

Further, besides being employed in solitary treatment regimes, Cur has also been employed in combinatorial treatment regimes. To this end, a report investigated the combined action of Cur and irinotecan on colorectal cancer (CRC) cells (LOVO cell) using in-gel protein digestion and MALDI-TOF/TOF MS [229]. Interestingly, it was found that out of a total of 54 protein spots differentially expressed, four exhibited protein–protein interactions. The cocktail could seemingly enhance the expression of protein disulphide isomerase (PDI) and peroxiredoxin-4 (PRDX4) which disarranged the formation and reduction of disulphides, which consequently leads to enhanced apoptotic responses in LOVO cells. The authors speculated that Cur may lead to the suppression of glutathione S-transferase Mu 5 (GSTM5) expression that helps in enhancing the lethal effect of irinotecan. Another study concluded that Cur enhanced the effect of irinotecan against CRC cells through ROS generation and activation of the Endoplasmic Reticulum (ER) stress pathway. Proteomic analysis through MALDI-TOF/TOF MS revealed 11 repeated protein nodes, which are involved in intracellular calcium pathways, intracellular redox reaction pathways, and intracellular endoplasmic reticulum (ER) stress [281]. Furthermore, anti-metastasis activity of Cur, ginsenoside 20 (S)-Rg3, and oxaliplatin were comparatively evaluated using proteomic analysis in isogenic primary (SW480) and metastatic colon (SW620) cell lines. This combinational therapy demonstrated the suppressive effect of all three bioactive substances on fatty acid synthase and histone H4 expression. There was a significant reduction in migratory activity of SW620 cells, which suggests that theyteffectively retards cell migration in colon cancer [230].

Cumulatively, it is reasonable to envisage that all these proteomic studies provides a better understanding of the underlying intricacies for the anticancer potential of Cur.

#### 2.1.3. Proteomic Studies to Explore Its Potential against Various Other Disease Pathologies

Accumulating evidence has highlighted the intriguing role of Cur against atherosclerosis [240,282]. In order to understand the molecular intricacies, the proteomic analysis ofmonocyte/macrophage-like cells (RAW264.7) cultured in the presence of Cur revealed considerable alteration in the proteome profile. This included increased expression of cytochrome b5 (cb5), ATP synthase, non-muscle myosin alkali light chain, and MHC class II protein moieties in RAW264.7 cells. On the other hand, decreased expression for various key players such as ran binding protein (RanBP)-1, phosphodiesterase 4D, eukaryotic initiation factor 3 (elF-3), nucleophosmin, vimentin, and heterogeneous nuclear ribonucleoprotein F (Hnrpf) protein were found as well. These data indicated the involvement of Cur in a myriad of functions including modulation of cell inflammation, reduction in the accumulation of intracellular cholesterol, antioxidant activity, and inhibition of cholesterol transport in RAW264.7 cells. Collectively, this study conclusively supports the anti-atherosclerosis mechanism of Cur seemingly through regulation of the accumulation of intracellular cholesterol levels and its transport [240].

Furthermore, Cur effectiveness and beneficial properties have also been investigated in weight management employing proteomic approaches. As a matter of fact, browning of white adipose tissue is an intriguing approach to combat obesity by enhancing energy expenditure. To demonstrate the protein involved in the fat-browning effect, proteomic modifications were analyzed in cultured white adipocytes under Cur treatment. Analysis through 2-DE combined with MALDI-TOF-MS revealed differential expression of ~58 protein spots among the control and Cur-treated adipocytes; out of which, hormone-sensitive lipase (HSL), an interacting partner of another two browning markers, uncoupling protein 1 (UCP1) and Peroxisome proliferator-activated receptor gamma coactivator 1-alpha (PGC-1α) were found to be prominently associated with the browning phenotype. Overall, this study suggests that Cur induces the HSL level in white adipocytes, which in turn induces fat browning [241].

Further, as a matter of fact, pulmonary fibrosis is an impaired fibrinolytic system that is associated with inflammation of the alveoli, which thereby leads to deposition of extracellular matrix (ECM) components and myofibroblasts. In order to investigate the therapeutic potential of Cur against pulmonary fibrosis disease progression, Gouda et al. employed high throughput Q-Orbitrap MS technology. In their study, basically C57BL/6 mice were injected with Bleomycin (BLM), followed by Cur treatment for 24 and 48 h time intervals [242]. The results of proteomic analysis revealed fascinating outcomes; it was found that BLM-exposed mice showed gradual weight loss and altered lung morphology. On the other hand, these symptoms were considerably reversed following Cur treatment. The proteomic analysis suggested strong interaction of Cur with p53, PAI-I, and uPA proteins as an expression of IL-17A-mediated inflammation in the impairment of the p53-fibrinolytic system and alveolar epithelial cell (AEC) apoptosis, which is a critical pathophysiological hallmark of pulmonary fibrosis. The results suggested that Cur could act as a potential therapeutic candidate to target the fibrinolytic system during pulmonary fibrosis, alongside its protective role against the progression of pulmonary fibrosis [242].

Further, accumulating evidence has highlighted the intriguing role of Cur as an effective therapeutic agent against various neurodegenerative diseases [243,283,284,285]. To this end, Urbani and group highlighted an intriguing molecular investigation of the main proteome rearrangements involved in the cellular response to Cur in human neuroblastoma cells sensitive to cisplatin and its resistant counterpart through shotgun proteomics analysis. Interestingly, the comparative proteomics analysis revealed that 66 proteins were differentially expressed following Cur treatment in sensitive cells. On the other hand, 32 proteins were differentially expressed in resistant treated cells. Further, gene ontology studies revealed that proteins involved in cellular assembly and organization, biosynthesis, and glycolysis were downregulated following Cur treatment. Moreover, proteome changes were also associated with cell cycle arrest in the G2/M phase and accumulation of polyubiquitinated proteins. As a matter of fact, the polyubiquitination of proteins influences a wide range of cellular processes; thus, the inhibition of the ubiquitin–proteasome system might be the major way through which Cur performs its multifactorial effects [243].

Further, studies have shown that Cur protects against Alzheimer’s disease plausibly via binding to sensile plaques and thereby inhibiting plaque pathology, Aβ plaque aggregation, and reduction in amyloid levels [38,39,41]. Moreover, Cur has been demonstrated to attenuate parkinsonism as well, seemingly through modulation of human α7-nicotinic acetylcholine receptor (α7-nAChRs) [40,286].

Further, Cur has been found to be an intriguing agent to be used following cataract surgery. Basically, a cataract is an opacification (cloudy appearance or opaqueness) of the eye lens that causes a decrease in vision. After cataract operation (i.e., lens replacement), some patients develop the symptom of faded vision. The condition occurs due to posterior capsular opacification (PCO) after cataracts removal. Unfortunately, it is a prevalent side effect following lens replacement. Therefore, attenuation of proliferation of lens epithelial cells (LECs) could plausibly prevent and/or repress PCO. To this end, Hu et al., used MS to investigate the inhibitory action of Cur against the proliferation of human lens epithelial B3 cells (HLE-B3). The proliferation of HLE-B3 cells was induced through administration of recombinant human basic fibroblast growth factor (rhbFGF), followed by treatment with Cur (20 mg/L). Interestingly, the results of this study showed that Cur acts as an effective inhibitor of the HLE-B3 cell proliferation induced by rhbFGF [244]; this plausibly endorses its effectiveness in management of post-cataract complications.

As already mentioned, alternative and/or complementary medicine have shown intriguing potential against various disease pathologies including cancer. Albeit often perceived as innocuous, these phytochemicals can seemingly interact with various metabolic enzymes including cytochrome P450s (CYPs), UDP glucuronosyl-transferases (UGTs), and drug transporters (e.g., P-gp, MRP, OATP). These intricacies have highlighted the need to understand potential phytochemical–molecular interactions To this end, a critical study evaluated the effect of oral Cur on intestinal uridine diphosphate glucuronosyltransferase (UGTs) expression in healthy volunteers aged between 40–80 years through LC-MS/MS [245]. In this study, all volunteers consumed daily curcuminoid extract (4 g) for 30 days. Interestingly but not surprisingly, proteomic data analysis did not reveal any significant differences in rectal mucosal UGT concentrations before and after Cur administration. Concomitantly, this study indicates that daily Cur use is unlikely to alter colonic UGT expression, especially in colon cancer [245]. Nevertheless, whether this is true with other forms of cancer as well seemingly requires further investigations to truly understand the Cur–metabolic enzyme interactions.

### 2.2. Proteomic Approaches to Understand the Function of Genistein as Therapeutic Intervention

Gen embodies broad range of vital properties, including antioxidant, anti-inflammatory, anti-microbial, anti-cancer, and so on [45,151,152,153]. As a matter of fact, a type of breast cancer classified as triple-negative breast cancer (TNBC) is estrogen receptor-negative, progesterone receptor-negative, and Her2-negative. Overall the survival, whether in early-stage or advanced disease stage, is poor in TNBC patients. Unfortunately, there are shortage of targeted therapies for TNBC. Of note, Gen is known for its estrogenic potential and accumulating data has highlighted its anticancer potential in breast cancer. To explore more about the anticancer potential of Gen against TNBC; Fang et al. performed phosphoproteomics studies. Interestingly, they identified approximately 5445 phosphorylation sites on 2008 phosphoproteins following Gen treatment. Further, bioinformatics analysis indicated the presence of 332 Gen-regulated phosphorylation sites on 226 proteins. Thus, proteomic data revealed that Gen may be involved in the critical cell cycle processes, including DNA replication, cohesin complex cleavage, and kinetochore formation. Additionally, Gen potentiates the activation of DNA damage responses, such as activation of ataxia telangiectasia serine/threonine-protein kinase (ATR) and breast cancer susceptibility gene 1 (BRCA1) complex. Conclusively, this phosphoproteomics study revealed the complex role of Gen in the regulation of the cell cycle and DNA damage response [251].

Another study explored concentration-dependent anticancer activity of Gen against SNU-449 cells [253]. The study reported its apoptosis-associated signature characteristics including involvement in caspase-3 activation as well as DNA fragmentation. Proteomics analysis revealed the involvement of antioxidant protein, thioredoxin-1, in Gen-induced apoptosis. Of note, thioredoxin-1 levels were found to be downregulated following Gen treatment, resulting in increased accumulation of ROS intracellularly. Moreover, Gen potentiated activation of different signalling mediator proteins including c-Jun N-terminal kinases (JNK), apoptosis signal-regulating kinase 1, and p38. Interestingly, prior treatment of JNK and p38 inhibitors can considerably abolish Gen-induced apoptotic responses. Concomitantly, the study concluded that Gen induces apoptotic responses in SNU-449 cells were plausibly through reduction in the thioredoxin-1 concentration and activation of JNK, apoptosis signal-regulating kinase 1, and p38 kinase thereof [253].

Another study explored the proteomics alterations in rat mammary glands following Gen treatment. Basically, the female rats were exposed to Gen by different routes through lactating dams and thereafter, the mammary glands were collected at day 21 and 50 post treatment and subjected to proteomic studies. The study revealed alteration in expression of ~23 proteins. Wherein, proteins such as Annexin A2, Gelsolin, Phosphoglycerate kinase-1 (P1), protein disulfide isomerase A3 (PDIA3), vascular endothelial growth factor receptor 2 (VEGF-R2), and epidermal growth factor receptor (EGF-R) were further validated through immunoblot assay. Of note, differential expressions of these proteins at different time points were found; for instance, expression of annexin A2 were found to be increased at the 21st day and reduced at the 50th day. On the other hand, PGK1 levels remained unchanged at the 21st day but decreased around the 50th day. Similarly, fetuin B expression was unaltered until the 21st day but increased on the 50th day, whereas the expression of VEGF-R2 and EGF-R were decreased at the 50th day in the mammary gland [259].

One more study by Wang et al. focused on the protein biomarkers for both effectiveness and susceptibility to breast cancer in blood and urine of prepubertal girls exposed to selected environmental chemicals with high urine concentrations of Gen, BPA, mono-ethyl hexyl phthalate (MEHP), and mono-benzyl phthalate (MBzP). Proteomics data suggested that the differentially regulated cancer-related proteins in girls with high concentrations of BPA and Gen corelated well with previously reported functions of BPA in carcinogenesis and of Gen in mammary cancer prevention, respectively [261].

Further, another proteomics study was performed to identify the prospective protein biomarkers and their association with carcinogenesis upon exposure to bisphenol A (BPA, a cancer-causing agent) and/or Gen to prepubertal rats. The results of the study suggested that Gen pre-exposed rats showed decreased expression of matrix metalloproteinase-3 (MMP3), rho associated coiled-coil containing protein kinase 2 (ROCK2), VGF nerve growth factor inducible, and Alpha-1 antitrypsin (SERPINA1), whose overexpression has previously been associated with carcinogenesis in various types of human cancers. Similarly, three tumor suppressor proteins (UCH1, SETD2, and PTPRK) were found to be upregulated in Gen-exposed rats. Thus, decreased expression of carcinogenesis inducing protein and increased expression of a tumor suppressor protein seems to be responsible for the chemoprotective action of Gen in animal model [262].

Gen treatment has also been employed against various other human disease pathologies such as cardiovascular diseases, liver fibrosis, and so on. Due to its potential role in the cardiovascular system, Gen has been reported to be associated with a lower blood pressure condition either directly or indirectly. It has been shown that Gen considerably attenuates vascular contraction; thereby regulating vascular tone and blood pressure plausibly via regulation of myosin light chain (MLC) phosphorylation, mediated through myosin light chain kinase (MLCK) or the RhoA signaling cascade. Further, it has been highlighted that oral consumption of Gen altered the level of the various cytoskeletal and contractile proteins in ovariectomized female mice, and also increased the phosphorylation of MLC. This study, contrary to others, suggests that Gen does not inhibit the MLCK or RhoA pathway [263]. Thus, it necessitates further investigation to fill in these gaps and plausibly resolve these discrepancies.

It is widely acknowledged that after menopause, estrogen elevates the risk of cardiovascular disease in women. Therefore, a novel strategy is to replace estrogens with alternative hormones like phytochemical Gen that acts as a natural estrogenic compound and reduces the effect. To this end, a 2-DE/ESI-LC-MS approach was used to investigate the effects of a dietary supplement with the phytoestrogen Gen on the cardiac proteome pattern for young, adult, and castrated male and female mice. Basically, the protein species diversification and their alteration were studied following Gen intake. The authors noticed substantial effect on the relative abundance of estrogen receptors, even through oral consumption. This investigation revealed expression of several fatty acid metabolism associated enzymes, and interestingly, their transcriptional regulators varied in male and female mice at both the transcriptional as well as at the protein level. Moreover, they also noticed that Gen increased the protein levels in male mice, which was found to be closely associated with oxidative phosphorylation and generation of ROS. On the other hand, in female mice, Gen elevates the level of two isoforms of LIM (LIN-11, Isl-1, and MEC-3) domain-binding protein and one isoform of desmin, which is associated with cardiac hypertrophy [264]. Taken together, this research endeavor revealed a complex influence of Gen on the proteome of the murine heart and warrants further investigations for a better understanding of the influence of Gen in myocardial pathology. In analogy, another study further investigated the effects of fixed concentrations of Gen on HL-1 cardiomyocyte cells. They noticed that various proteins were differentially expressed upon treatment with 1 μM and 50 μM Gen, and that both concentrations of Gen impacted the regulation of ATPase activity and glucose catabolic processes. Nevertheless, at lower concentrations, Gen significantly influences the heat shock proteins and anti-apoptotic responses. Furthermore, a higher concentration reduces glycolytic proteins and antioxidant enzymes, which consequently leads to energy depletion and apoptotic responses, making the cardiomyocytes potentially more susceptible [256].

It is widely envisaged that liver fibrotic conditions ensue when healthy tissue of the liver becomes scarred, which can turn into chronic liver diseases at later stages. Researchers have evaluated the anti-fibrotic activity of Gen on gastric cancer cell line AGS by proteomic analysis using combination therapy including Gen and other phytochemicals such as taurine, epigallocatechin, and gallate. A proteomics study revealed the involvement of these phytochemicals in the improvement of liver function. A total of 89 protein alterations were reported, out of which four differentially expressed proteins (Tpi1, Txn1, Fgb, and F7) were involved in the glycolysis pathway, coagulation cascade pathway, and antioxidant defense system. Further investigation revealed reduced expression of aspartate transaminase (AST), alanine aminotransferase (ALT), transforming growth factor-β1 (TGF-β1), and collagen I, and increased expression of superoxide dismutase (SOD), total antioxidative capacity (T-AOC), and glutathione peroxidase (GSH-Px). This study suggested the use of combination therapy as an alternative treatment against liver fibrosis [265]. Furthermore, another study provided combinational therapy to rats followed by proteomic analysis of collected liver tissue. Intriguingly, proteomic alteration of 115 proteins was detected, in which 31 proteins were found to be downregulated, whereas 84 were differentially up-regulated. Out of these, three proteins including Txn1, Ctsd, and Cdk4 were selected for further investigation through real-time PCR and Western blotting. Conclusively, this study suggested the significant correlation of these proteins with liver fibrosis and also clarified the role of combination therapy as a potential intervention for the treatment of liver fibrosis [266].

Besides these, Gen has been found to be a promising candidate against neurodegenerative diseases as well. Interestingly, it has displayed antioxidant potential via annihilation of free radicals [287] and amelioration of antioxidant enzyme activity [288], and thus plausibly leads to prevention and/or treatment of Alzheimer’s disease pathology [47]. It has been shown to antagonize the toxicity of amyloid β-protein (Aβ), and thereby could be useful as an intriguing neuroprotective agent. It has been demonstrated that Gen considerably decreases Aβ production seemingly through inhibition of Beta-site (βsite) amyloid precursor protein (APP) cleaving enzyme 1 (BACE1) [48,49]. It also counteracts the progression of Parkinson’s disease through several intricate mechanisms [50].

Collectively, as more and more data are gleaned, proteomics studies will be highly instrumental in broadening our understanding regarding the molecular intricacies of Gen-based therapeutic interventions.

### 2.3. Proteomic Approaches to Understand the Function of Tan IIA as Therapeutic Intervention

Tan IIA has been widely known for its therapeutic potential against myriads of disease pathologies [6,11]. Interestingly, but not surprisingly, analysis of its therapeutic potential from a proteomic perspective would be highly instrumental for a comprehensive understanding of their mechanisms of action. Accordingly, several reports have highlighted the molecular intricacies underlying the therapeutic potential of Tan IIA against various disease pathologies employing proteomic studies. To this end, Pan et al. focused on the changes in the proteome of HeLa cancer cells treated with Tan IIA using MALDI-TOF analysis. Their proteomic data revealed the alteration of 12 differentially regulated proteins in the HeLa cancer cells following treatment with Tan IIA. The expression levels of proteins such as heat shock protein 27 (HSP27), vimentin, tubulin, and vinculin that play an important role in signal transduction pathways, energy metabolism, motility and microtubule assembly were found to be considerably modified. They proposed that these protein molecules could be related to HeLa cell growth inhibition [268]. Interestingly, the authors envisaged that the plausible contributions of these proteins to the cytotoxicity of Tan IIA seemingly provides intriguing opportunities for the development of Tan IIA-based cancer therapeutics.

In another study, Long et al. performed label free nano-LC-MS/MS-based proteomic analysis to identify the proteome changes in hepatocellular cancer cells (MHCC97-H) in response to Tan IIA treatment. They identified ~41 significantly altered proteins; most of them were associated with various cellular functions such as microtubule movement, stress resistance, cytoskeletal organization, and translational and transcriptional regulation. Furthermore, they proposed that these proteins could play an important role in hepatocellular cancer tumorigenesis [269]. Further, in a similar kind of study, Lin et al. performed iTRAQ-based proteomics studies along with RNA-seq transcriptomics studies and showed that the suppression of AGS gastric cancer cell growth was seemingly due to alteration of glucose metabolism. They further identified ~102 altered signature proteins. Gene enrichment analysis led to the discovery of the role of various dysregulated proteins in alteration of several key cellular functions such as apoptosis, cell cycle, DNA damage, carbohydrate metabolism, and cytoskeleton reorganization functions. In particular, they identified down-regulation of L-lactate dehydrogenase B chains and glucose-6-phosphate isomerase proteins. These results suggested that Tan IIA plays a key role in the blocking of glucose metabolism, thereby inhibiting cell proliferation [270]. Similarly, 2-DE proteomic analysis of MG-63 osteosarcoma cancer cells with and without treatment with Tan IIA along with other two anticancer agents, i.e., Ginsenoside Rg1 and Cinnamic acid, identified prohibitin as a dramatically down-regulated protein in the nuclear matrix in the treated cells. Authors validated this protein using Western blot and immunogold electro-microscopy analysis. They also stated that prohibitin acts as a molecular chaperone and regulates several oncogenes as well as tumor suppressor genes, thereby playing an important role in cancer treatment [272]. Further, Yin et al. performed phosphoproteomics analysis for the Tan IIA treated AGS gastric cell line through a label free proteomic approach using linear ion trap (LTQ)-Orbitrap. In this study, they identified HSP27 phosphorylation at serine 82 in response to Tan IIA treatment. Moreover, they reported that phosphorylation of HSP27 leads to the production of ROS in the gastric cancer cells, emphasizing its importance in cancer cell apoptosis [273].

Further, other proteomic studies have highlighted the molecular intricacies of Tan IIA against various other human diseases such as liver fibrosis, cardiac failure, and nephropathy. To this end, Pan et al. used a 2-DE-based proteomic approach followed by MALDI-TOF analysis to identify the global proteome modifications of HSC-T6 cell line treated with Tan IIA. In this study, they found 13 proteins with altered expression patterns and among these proteins, prohibitin showed an upregulated pattern. They further validated this study using Western blotting analysis, while knockdown studies revealed its role in attenuation of apoptosis in liver fibrosis [271]. In another study, Yan et al. studied the proteomic alterations in transverse aortic constriction (TAC)-mediated cardiac failure and the cardio protective function of Tan IIA using label free Liquid chromatography-matrix-assisted laser desorption/ionization mass spectrometry (LC-MALDI-MS) proteomic approach. They identified 44 differentially regulated proteins common in control vs. saline-TAC and Tan IIA-TAC vs. saline-TAC. Most of these proteins were associated with mitochondrial function of myocardial cells. Proteins such as carnitine palmitoyl transferase I (CPT-1) and glucose transporter type 4 (GLUT-4) were downregulated when the myocardial cells were treated with TAC, whereas their expression levels recovered to normal after treatment with Tan IIA. These proteins are involved in the metabolic activity of mitochondria by regulating free fatty acids and glucose transfers [276]. In a similar study, Liu et al. performed the 2-DE-based proteomic approach followed by MALDI-TOF MS/MS analysis to identify the protein alterations in doxorubicin nephropathy vs. Tan IIA mediated kidney protection. They identified 17 altered proteins in the control male Kunming (KM)-mice as compared to doxorubicin-treated mice. Twelve of these proteins exhibited downregulation while five of them showed upregulation. Further analysis revealed that out of these twelve downregulated proteins, eight were significantly reversed when doxorubicin-treated male KM-mice were further treated with Tan IIA. Similarly, out of five upregulated proteins, expression of three proteins were reversed following treatment with Tan IIA. These proteins were found to be mainly involved in various cellular activities such as oxidative stress, protein synthesis, cytoskeleton synthesis, etc. [278].

Furthermore, Tan IIA has shown intriguing neuroprotective potential against Alzheimer’s, Parkinson’s, and multiple sclerosis [289,290]. Tan IIA has displayed inhibition of acetylcholinesterase and butyrylcholinestrase, which cause degradation of acetylcholine and thus disrupts cholinergic neurotransmission [57,58]. Interestingly, it has shown promising activity as a learning and memory booster and neuroprotectant against the Aβ plaque- and APP-induced AD symptoms in rodents. Moreover, the neuroprotective effect of Tan IIA is exerted by its anti-inflammatory effect in the brain as indicated by attenuation of astrocytic and microglial activation, proinflammatory cytokines (TNF-α, IL-1β, and IL-6) production, and NF-κB signaling in the cortex and hippocampus in the brains of mice [59]. Likewise, Tan IIA has been demonstrated to ameliorate 6-hydroxydopamine (6-OHDA)-induced dopaminergic neuronal loss seemingly through activation of the NF-E2-related factor 2 (Nrf2)–antioxidant response element (ARE) signal transduction pathways [60].

In summation, it is envisaged that proteomics studies have been highly instrumental in broadening our understanding regarding the molecular intricacies of Tan IIA-based therapeutic interventions.

## 3. Conclusions

Accumulating evidence has highlighted the therapeutic potential of various phytochemicals for the treatment and management of various disease conditions. It is reasonable to argue that the pharmaceutical industry is seeking a gradual shift from chemically derived drugs to phytochemically derived drugs. Numerous proteomics-based studies have highlighted the promising effects of Cur, Gen, and Tan IIA against various pathological conditions including microbial infections, metabolic disorders, cancer, neurodegenerative diseases, and soon; and provided a molecular rationale for their therapeutic potentials. It is envisaged that proteomics as a technology is evolving at a fast pace; henceforth, with the continued technical advancements, it would be highly instrumental in orchestrating much deeper insights in phytochemical-based therapeutic interventions.

## Figures and Tables

**Table 1:** Representative table highlighting various phytochemicals along with their pharmacological properties and therapeutic importance.

S. No.	PhytochemicalName	Appearance	Chemical Structure	Molecular Weight, Chemical Formula, Pubchem CID, and IUPAC Name	Prospective Pharmacological Properties
1.	Curcumin	Bright yellow-orange	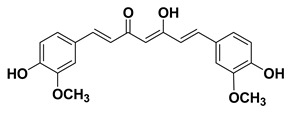	Mol wt: 368.38 g/mol Chemical formula: C21H20O6 Pubchem CID:969516 IUPAC name: 1,7-Bis(4-hydroxy-3-methoxyphenyl)hepta-1,6-diene-3,5-dione	It acts as an antioxidant [25], anti-inflammatory [26] anti-bacterial [27], anti-fungal [28] antiviral [29], and anti-neoplastic agent [30]. It exhibits phototoxic and photodynamic activities [31,32,33], acts as a cyclo-oxygenase inhibitor [34], lipoxygenase inhibitor [35], iron chelator [36], immunomodulator [37], and neuroprotective agents [38,39,40,41].
2.	Genistein	Yellow	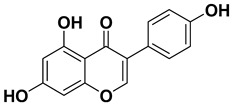	Mol wt: 270.24 g/mol Chemical formula: C15H10O5 Pubchem CID: 5280961 IUPAC name: 5,7-dihydroxy-3-(4-hydroxyphenyl)chromen-4-one	It has antioxidant [42], anti-inflammatory and immunosuppressive activities [43]; it acts as anti-microbial agent [44], it embodies anti-carcinogenic and anti-metastatic properties [45]. It also acts as a phytoestrogen and a protein tyrosine kinase inhibitor [46], and neuroprotective agent [47,48,49,50].
3.	Tanshinone IIA	Red	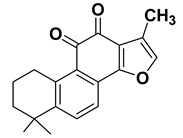	Mol wt: 294.3 g/mol Chemical formula: C19H18O3 Pubchem CID: 164676 IUPAC name: 1,6,6-trimethyl-8,9-dihydro-7H-naphtho [1,2-g] [1]benzofuran-10,11-dione	It embodies antioxidant [51], anti -inflammatory [52], anti-microbial [53], anti-cancer [54], anti-angiogenic [55], and anti-adipogenic properties [56], and embodies neuroprotective properties [57,58,59,60].
4.	Allicin	Slightly yellow	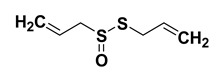	Mol wt: 162.3 g/mol Chemical formula: C6H10OS2, Pubchem CID: 65036 IUPAC name: 3-prop-2-enyl sulfinyl sulfanyl prop-1-ene	It embodies free radical scavenging properties, viz., anti-oxidant [61], anti-bacterial [62,63,64], anti-fungal [63], and anti-viral properties [65]. It exhibits antihypertensive [66] and neuroprotective properties [67] and acts as hypo-lipidemic and hypo-glycemic [68,69] and anti-cancer agent [70,71,72].
5.	Eugenol	Pale yellow	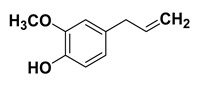	Mol wt: 164.2 g/mol Chemical formula: C10H12O2 Pubchem CID: 3314 IUPAC name: 2-methoxy-4-prop-2-enyl phenol	It embodies antioxidant [73], anti-inflammatory [74], anti-microbial [75], anti-tumor [76,77,78,79], anti-mutagenic [76,77,78,79], anti-allergic [80], antipyretic [81] and analgesic characteristics [80]. It is a 5-lipoxygenase inhibitor [76], anti-hypercholesterolemic, and anti-atherogenic potential [82], antidiabetic [83], antiparasitic [84], and anti-leishmanial agent [85].
6.	Apigenin	Yellow	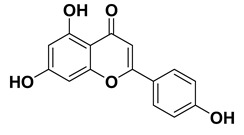	Mol wt: 270.24 g/mol Chemical formula: C15H10O5 Pubchem CID: 5280443 IUPAC name: 5,7-dihydroxy-2-(4-hydroxyphenyl) chromen-4-one	It is an antioxidant [86], anti-inflammatory [87,88,89], anti-bacterial [90], anti-viral [91], anti-cancer [92], chemo-preventive agent, anti-invasive [92,93], and antidiabetic agent [94,95,96] and embodies neuroprotective [97] and vasodilatory action [98].
7.	Lycopene	Bright Red	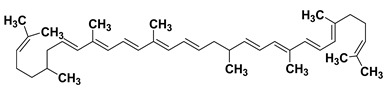	Mol wt: 536.873 g/mol Chemical formula: C40H56, Pubchem CID: 446925 IUPAC name: 2,6,10,14,19,23,27,31-octamethyldotriaconta-2,6,8,10,12,14,16,18,20,22,24,26,30-tridecaene	It is an antioxidant [99], anti-inflammatory [100], anti-microbial [101], anticancer [102], radiation-protective agent [103], and embodies cardioprotective [104] and neuroprotective properties [105].
8.	Anthocyanin	Red, purple, and blue	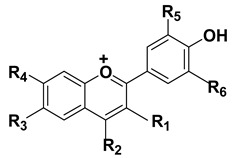	Mol wt: 207.24724 g/mol, Chemical formula: C15H11O+, Pubchem CID: 145858 IUPAC name: 2-phenylchromenylium	It embodies antioxidant [106], anti-inflammatory [107], anti-microbial [108], antiviral [109], and anticancer properties [110]; it embodies hypouricemic and nephroprotective effects [111]. It acts as a cyto-protective [112] and neuroprotective agent [113].
9.	Capsaicin	Crystalline white	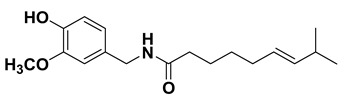	Mol wt: 305.4 g/mol, Chemical formula: C18H27NO3 Pubchem CID:1548943 IUPAC name: (E)-N-[(4-hydroxy-3-methoxyphenyl) methyl]-8-methylnon-6-enamide	It act as an antioxidant [114], anti-inflammatory [115], anti-bacterial [116], anti-fungal [117], anti-viral [118], and anticancer agent [119]. It act as an analgesic [120], gastroprotective [121], anti-obesity [122], and antipruritic agent [123]. It embodies anti-proliferative and pro-apoptotic properties against cancer [124].
10.	Shogaols	Bright yellow	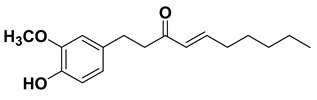	Mol wt: 276.376 g·mol^−1^, Chemical formula: C17H24O3, Pubchem CID: 5281794 IUPAC name: (E)-1-(4-hydroxy-3-methoxyphenyl)dec-4-en-3-one	It has been found as an antioxidant [125,126,127], anti-inflammatory [128,129,130,131], anti-bacterial [132], anti-fungal [133], anti-viral [134], anticancer, and chemo-preventive agent [135] with anti-emetic and anti-thrombotic properties [136].

## Abbreviations

ACTC1	Alpha cardiac muscle protein
AEC	Alveolar epithelial cells
ALT	Alanine aminotransferase
AST	Aspartate transaminase
ATR	Ataxia telangiectasia serine/threonine-protein kinase
BLM	Bleomycin
BPA	Bisphenol A
BRCA1	Breast cancer susceptibility gene 1
BTF3	Basic transcription factor 3
CPT-1	Carnitine palmitoyl transferase I
CRC	Colorectal cancer
CRK2	Cysteine-rich receptor-like protein kinase
Cur-P	Curcumin probe
CUR/CDP	Curcumin/β-cyclodextrin polymer
2-DE	Two-dimensional gel electrophoresis
2D-DIGE	Two-dimensional differential gel electrophoresis
2-DE/ESI-LC-MS	Two-dimensional liquid chromatography/electrospray ionization mass spectrometry
DJ-1 (PARK7)	Protein deglycase DJ-1, also known as Parkinson disease protein 7
DMSO	Dimethyl sulfoxide
ECM	Extracellular matrix
eEF1	Eukaryotic elongation factor 1
EGF-R	Epidermal growth factor receptor
eIF2	Eukaryotic Initiation Factor 2
elF-3	Eukaryotic initiation factor 3
ER	Endoplasmic reticulum
FN-1	Fibronectin-1
FtsZ	Filamenting temperature-sensitive mutant Z
GBM	Glioblastoma multiforme
GLUT-4	Glucose transporter type 4
GSH-Px	Glutathione peroxidase
GSN	Gelsolin
GSTM5	Glutathione S-transferase Mu 5
HNRPF	Heterogeneous nuclear ribonucleoprotein F
HSC71	Heat shock cognate 71
HSL	Hormone-sensitive lipase
HSP27	Heat shock protein 27
IPA	Ingenuity pathways analysis
iTRAQ	Isobaric tags for relative and absolute quantitation
JNK	c-Jun N-terminal kinase
KM-mice	Kunming mice
LC-MALDI-MS	Liquid chromatography-matrix-assisted laser desorption/ionization mass spectrometry
LC-MS/MS	Liquid Chromatography with tandem mass spectrometry
LECs	Lens epithelial cells
LFQ	Label free quantitation
LTQ-Orbitrap	Linear ion trap-Orbitrap
MALDI-TOF/TOF-MS	Matrix-assisted laser desorption ionization tandem time-of-flight mass spectrometry
MBzP	Mono-benzyl phthalate
MEHP	Mono-ethyl hexyl phthalate
MLC	Myosin light chain
MLCK	Myosin light chain kinase
MMP3	Matrix metalloproteinase-3
MRM	Multiple reaction monitoring
MS	Mass spectrometry
mTOR	Mammalian target of rapamycin
NPM1	Nucleophosmin
PCO	Posterior capsular opacification
PDI	Protein disulphide isomerase
PDIA3	Protein disulfide isomerase A3
PGAM1	Phosphoglycerate mutase 1
PGC-1α	Peroxisome proliferator-activated receptor gamma coactivator 1-alpha
PGK1	Phosphoglycerate kinase-1
PRDX4	Peroxiredoxin-4
PRDX6	Peroxiredoxin-6
Q-Orbitrap MS	Quadrupole-Orbitrap Mass Spectrometer
RanBP-1	Ran binding protein-1
rhbFGF	Recombinant human basic fibroblast growth factor
ROCK2	Rho associated coiled-coil containing protein kinase 2
ROS	Reactive oxygen species
SILAC	Stable isotope labeling by amino acids in cell culture
SOD	Superoxide dismutase
STAT3	Signal transducer and activator of transcription 3
SWATH-MS	Sequential window acquisition of all theoretical mass spectra
SDS-PAGE	Sodium dodecyl-sulfate polyacrylamide gel electrophoresis
T-AOC	Total antioxidant capacity
TAC	Transverse aortic constriction
Tan IIA	Tanshinone IIA
TGF-β1	Transforming growth factor-β1
TMT	Tandem mass tags
TNBC	Triple-negative breast cancer
TPI	Triosephosphate isomerase
UCP1	Uncoupling protein 1
UGT	Uridine diphosphate glucuronosyltransferase
VEGF-R2	Vascular endothelial growth factor receptor 2
VHSV	Viral hemorrhagic septicemia virus
